# Optimal Location through Distributed Algorithm to Avoid Energy Hole in Mobile Sink WSNs

**DOI:** 10.1155/2014/894018

**Published:** 2014-05-07

**Authors:** Li Qing-hua, Gui Wei-hua, Chen Zhi-gang

**Affiliations:** ^1^School of Information Science and Engineering, Central South University, Changsha 410083, China; ^2^Institute of Technology, Lishui University, Lishui, Zhejiang 323000, China

## Abstract

In multihop data collection sensor network, nodes near the sink need to relay on remote data and, thus, have much faster energy dissipation rate and suffer from premature death. This phenomenon causes energy hole near the sink, seriously damaging the network performance. In this paper, we first compute energy consumption of each node when sink is set at any point in the network through theoretical analysis; then we propose an online distributed algorithm, which can adjust sink position based on the actual energy consumption of each node adaptively to get the actual maximum lifetime. Theoretical analysis and experimental results show that the proposed algorithms significantly improve the lifetime of wireless sensor network. It lowers the network residual energy by more than 30% when it is dead. Moreover, the cost for moving the sink is relatively smaller.

## 1. Introduction

Wireless sensor networks are a self-organizing network composed of random distribution of intensive, low-cost, integrated sensors with the function units of sensing module, data-processing module, and short-range wireless communication module. By the measurement the surrounding environment such as humidity, temperature, heat, infrared, sonar, radar, and seismic waves with a variety of built-in sensors to sensor network has achieved a full range of monitoring and controlling the physical world. It is an important integral part of long-term planning of next generation Internet and has already been widely used in the past few years [1].

Sensor nodes usually cannot be replaced or reallocated energy in wireless sensor network, and most applications need to ensure long-term monitoring of certain areas (most applications have prespecified lifetime requirements); for example, the application mentioned in [2] requires that the effective monitoring time for the network should be greater than 9 months to extend the life of sensor network and, thus, is of great significance.

However, research to improve the network life is of great challenges. There is a sensor network-specific “energy hole” phenomenon, which refers to premature death of those nodes in the hotspot. In multihop data collection sensor network, nodes near the sink have to suffer more routing load [3], so the energy consumption level is higher than nodes in other regions. This is known as the hotspot. Study shows that because of the impact of the energy hole, the network residual energy is as high as 90% [4] when the network is out of function.

Different from the general network with static sink, intelligent mobile robots can act as a mobile sink in the network to collect data. When the residual energy near the sink becomes small, sink repeatedly moves to the location with more abundant remaining energy so as to achieve a balanced energy consumption rate among the entire network, avoiding the energy hole and obtaining longer network lifetime.

Despite a lot of research on the mobile sink, different from previous studies, the main contribution of this paper is as follows.

1. Many researchers recognize the existence of hotspots near the sink, so mobile sink strategy is to move sink to the areas with the highest remaining energy in order to avoid energy hole near the sink and achieve the purpose of balance energy consumption. According to our study, when the sink is seated at different locations in the network, power consumption near sink is of different sizes. Energy consumption in different directions is also not the same. In many cases (especially when the sink is at the edge of the network) the power consumption in network-centric side of sink is much higher than the other side near the edge of the network. Meanwhile, on the side near network-centric, even energy consumption in regions far from the sink is higher than that of the near-edge side. Consequently, simple conclusion is that “areas near sink” which have higher energy consumption cannot effectively guide the design of sink mobility, and they are too general and difficult to be implemented in practice.

In this paper, through differential analysis, precise energy consumption of each node, for the first time, is given for arbitrary sink location in the network. This lays the foundation for mobile sink and energy balancing strategy.

(2) We propose distributed sink mobile strategy. Based on theoretical analysis of network energy consumption, it can estimate the power consumption of the network before sink moves to the new anchor and predict the optimal next location for sink according to the actual energy consumption of current network. In addition, previous studies fail to present a good solution to the cost of the sink migration. The distributed strategies proposed in this paper are different from previous studies. As the sequence of sink anchors does not affect the ultimate energy consumption and network lifetime, we usually do not move sink according to the calculated result. Instead, we compute a couple of anchors prior to moving and then traverse all the locations according to the minimum moving cost in each step in order to minimize the total cost.

The organization of this paper is as follows.  Section 2 introduces relevant research. The network model and the problem statement are presented in Section 3.  Section 4 introduces characteristics of data forwarding and energy consumption. It is the basis of theoretical research of our paper.  Section 5 describes distributed sink mobile strategy.  Section 6 discusses performance and experimental comparison.  Section 7 is a summary of the whole paper.

## 2. Related Work 

There are many existing researches handling energy hole problem. They can be divided into two categories based on the sink mobility: static sink network (for short, static sink) and mobile sink network (for short, mobile sink). Although this paper focuses on mobile sink, solutions to avoid energy hole in static sink can often be used here. Therefore, we first introduce related research with static sink.

- Analysis and evaluation model. Li and Mohapatra [5] first propose a mathematical model to analyze the energy hole problem. They assume that the network is uniformly distributed in a circle network and then analyze and compare performance of several existing strategies to avoid energy hole from the perspective of network traffic [6]. They point out that hierarchical structure and data compression mechanism are effective for alleviating the energy hole problem, increasing the rate of data collection, in turn, exacerbating the energy hole [5] and effectiveness of increasing number of the nodes is not obvious.
- Node density control strategy. The principle is that the higher energy consumption near the sink causes the energy hole problem, and therefore it is effective to deploy more nodes near the sink in order to reduce the data load of every node, alleviating the impact of energy hole. This is the so-called nonuniform distribution strategy. Another manifestation of node density control strategy is to deploy more nodes in the place where hotspots are possible to appear which is also effective. Such studies can be found in the literature [7–9].
- Adjustable sensing and communication range. The main principle of this type of strategy is that the node transmission power is adjustable; for example, Berkeley Motes node has 100 transmission power levels [10]. Node energy consumption is directly proportional to *α* power of transmission distance and therefore uses smaller transmission range in hotspots near sink and larger transmission range in regions far away from the sink which can achieve balanced energy consumption. This strategy can be combined with node density control strategy. For example, Hossain et al. [11] set the interval between nodes near the sink smaller so that the required energy to transmit data is small, set the interval between nodes far away from the sink larger, so the required energy to transmit data is larger. In this way, it can achieve balanced energy consumption among all of the nodes. Such studies can be found in the literature [12, 13].

The above discussion is under the situation where sink is stationary after deployment. However, with the development of intelligent robot, the research of mobile sink has attracted more and more attention [14]. The research in mobile sink can be summarized into the following categories.

- Relay nodes: such method is to use relay node in hotspot to avoid energy hole. Relay nodes can be both stationary and mobile. The role of mobile relay nodes is essentially similar with that of mobile sink. Related research can be found in the literature [15, 16].
- Single mobile sink: in this kind of networks there is only one sink, and it is movable. Luo and Hubaux [17] propose an early solution for mobile sink to solve the unbalanced energy consumption. As the sink can move, the nodes around it keep on changing over times, and thus it can avoid energy hole around the sink. The author proves that in a circular sensor network, putting sink in the center of a circle is the best way to save energy. Also, he claims that sink moving along the edge of the network can achieve minimum energy consumption.

Luo also puts forward a strategy that mobile sink moves along the anchor (anchor points) to collect data in [18]. The main idea is that when the sink stays in an anchor it collects data and gets the situation of energy consumption over the whole network in order to determine the interval to stay in every anchor.

As the energy consumption rate near the sink is relatively higher, [19] analogizes sink as “lawn” (mower) and the region with higher remaining energy as “grass” where relatively higher. So the strategy of moving the sink is the “lawn” (mower) moving to the region where “grass” is relatively higher repeatedly.

Reference [20] presents a mobile sink trajectory optimization algorithm and the main idea is as follows. At first, the mobile sink moves along a straight line and collects information about network data and energy consumption information. Mobile sink then adjust the trajectory using the latest information which collected in the process of data collection so that the mobile sink moves near the nodes in order to reduce the cost of data communication and thus to form an optimal trajectory of sink. The paper discusses random movement, forecast movement, and the network performance of different nodes of data collection patterns (passive, multihop, and limited multihop). Reference [21] sums up the target of mobile sink strategy into three categories: (1) minimizing average energy consumption; (2) minimizing largest energy consumption; (3) minimizing relative energy consumption and proposes corresponding mobile algorithms. More research on single mobile sink can be found in [22–24].

(c) Multiple mobile sinks: compared with single mobile sink, multiple mobile sinks will increase the cost of the network, but the network performance (network lifetime, network delays) can be greatly improved and, therefore, is subject to a wide range of research. However, mobile sinks require mutual cooperation and mutual coordination of movement between several sinks, and thus the study is more complicated than research of single mobile sink.

The latest research about multiple mobile sinks can be found in [24] which studies the optimization strategy of multiple mobile sinks and compares the situations of a number of stationary sinks and sinks moving along the hexagon surrounding. They find that sink mobility can significantly increase the network life, and the more points sink stays along the hexagon surrounding, the better balanced energy consumption the network will achieve. Mirela Marta, finally, proposes the distributed mobile optimization algorithm to maintain connectivity between the mobile sinks. Related research about multiple mobile sinks can also be found in [23, 24].

In addition, whether the research handles single or multiple mobile sink, they may adopt different strategies for data collection. It can be summed up in two circumstances. One is that mobile sink and static sink adopt the same type of data collection, using a static network sophisticated data collection strategies, such as single-hop, multihop, and subcluster. The other is to use different type data collection strategies from static sink, such as sensor nodes cache data so that the data is sent to sink when it arrives at vicinity of sensing, or the data is sent to a fixed regional cache, and then sink moves along the fixed region to collect data.

## 3. Network Model and Problem Description

**Figure 1:**
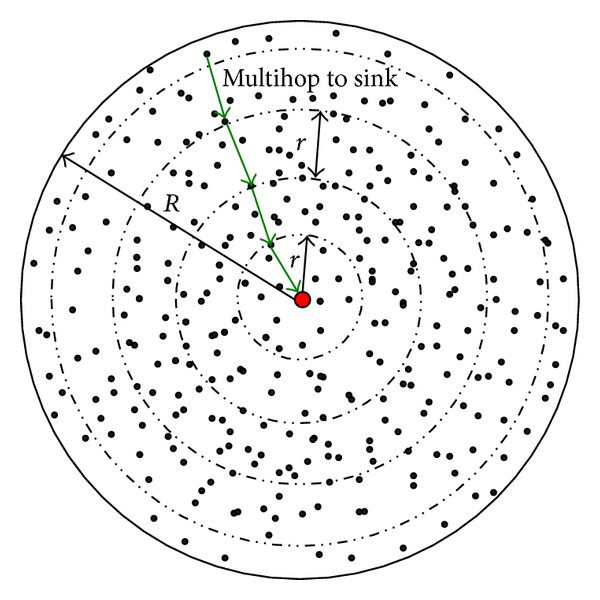
Network architecture model.

Network architecture model: we apply the module similar with [17, 19], a typical wireless sensor network for cyclical data collection and a circle with radius of *R*; see Figure 1. In this network, there are *n* nodes and, {*N*_0_, *N*_1_, *N*_2_, *N*_3_,…*N*_*n*_},   *N*_0_ stands for sink and it can move throughout the network; others represent work nodes and cannot move after initially deployed. Communication range of nodes is noted with *r*; the difference from general sensor networks is that the transmission range is adjustable, and nodes automatically adjust its communication range based on the distance between two nodes; for example, Berkeley Motes node has 100 transmission levels [7, 19]. Each work node will sense data in each cycle. We use the mature shortest path protocol for collecting data [8] and sending them to sink with multihop [8, 9].

Energy consumption model: we use typical energy consumption model; the cost of moving mobile sink is calculated according to formula (1), cost for sending data is calculated according to formula (2), cost for receiving data is calculated according to formula (3), and specific details can be found in the literature [14]:

(1) Esink(s)=sEe,

(2) Emember={lEelec+lεfsd2  if  d<d0,lEelec+lεampd4if  d>d0,

(3) ERx(l)=lEelec,

where *E*_elec_ stands for the energy loss of firing circuit. If the transmission distance is less than the threshold *d*_0_, power amplifier loss is based on free-space model; when the transmission distance is greater than or equal to the threshold value, it uses multipath attenuation model. *ε*_*fs*_, *ε*_amp_ represent the power for these two models' amplification, respectively. Energy for receiving *l* bit of data refers to formula (3). In this paper, the above specific parameters come from the literature [14].

*Problem Description*. For a given mobile sensor network shown in Figure 1, the problem can be described as follows: how to move and choose the anchors of mobile sink to maximize the network lifetime? Here we term the rounds of data collection till the first node dies as the network lifetime [7, 14]. 

## 4. Analysis Load of Node and the Tactor That Affects the Network Lifetime

### 4.1. Analysis Load of Node

When the sink moves to an arbitrary location such as (*x*_0_, *y*_0_), if it is able to calculate the data load of each node, it then will be easy to calculate the energy consumption of each node based on formulas (2) and (3) so as to learn energy consumption of the entire network. Therefore, this paper will compute data load for each sensor node when sink is located at arbitrary (*x*_0_, *y*_0_). To the best of our knowledge, this paper gives derivation of data load in the network. It is also the basis for sink strategy in this paper.

**Theorem 1**

Suppose the center of network be *O*(0,0), the sink moved to *A*(*x*_0_, *y*_0_), an optional sensor node *B* at (*x*_*b*_,*y*_*b*_), and the intersection point of *AB* extension with the network border is (*x*_*c*_,*y*_*c*_), then the data load for B node is as follows:

(4) Drx={(a−1−i)c+((a−1−i)(a+i)r/2)}(ir+c) if  D=ir+c, i∈{0⋯a}, c∈{b⋯r}  //receive  data,Dtx={(a−1−i)c+((a−1−i)(a+i)r/2)}(ir+c)+1 if  D=ir+c, i∈{0⋯a}, c∈{b⋯r}  //send  data,Drx={(a−i)c+((a+1+i)(a−i)r/2)}(ir+c) if  D=ir+c, i∈{0⋯a}, c∈{0⋯b}  //receive  data,Dtx={(a−i)c+((a+1+i)(a−i)r/2)}(ir+c)+1 if  D=ir+c, i∈{0⋯a}, c∈{0⋯b}  //send  data,where  R1=|AC|, α=⌊R1r⌋, R1=ar+b ∣ b≤r,D=|AB|=ir+c ∣ i∈{0⋯a},i=⌊Dr⌋,  c=D−ir, c∈{0⋯r},|AC|=(xc−x0)2+(yc−y0)2,|AB|=(xb−x0)2+(yb−y0)2.

**Proof**

**Figure 2:**
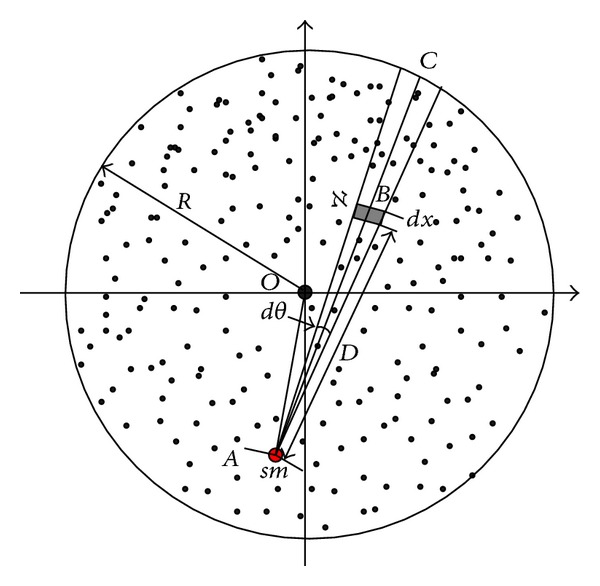
Network computing model.

This paper applies the shortest path routing protocol to transmit data to sink through multihop. For an arbitrarily node *B*(*x*_*b*_, *y*_*b*_), see Figure 2; *C* represents intersection point of *AB* extension with the network border, and the data load for *B* is the amount of data whose distance from *B* is integer multiple of *r* on line *BC*. First, we calculate the coordinates of *C*(*x*_*c*_, *y*_*c*_).

Equation of line *AB*:

(5) $$\begin{array}{c} y=\frac{y_{b}-y_{0}}{x_{b}-x_{0}}\left(x-x_{0}\right)+y_{0}. \end{array}$$

Equation of the circle:

(6) $$\begin{array}{c} x^{2}+y^{2}=R^{2}. \end{array}$$

Formula (5) can be simplified as

(7) $$\begin{array}{c} y=\frac{y_{b}-y_{0}}{x_{b}-x_{0}}\left(x-x_{0}\right)+y_{0}=\frac{y_{b}-y_{0}}{x_{b}-x_{0}}x-\frac{y_{b}-y_{0}}{x_{b}-x_{0}}x_{0}+y_{0}. \end{array}$$

Let *g*_1_ = (*y*_*b*_ − *y*_0_)/(*x*_*b*_ − *x*_0_), *g*_2_ = −(*y*_*b*_ − *y*_0_)/(*x*_*b*_ − *x*_0_)*x*_0_ + *y*_0_ = *y*_0_ − *g*_1_ *x*_0_

  
*y* = *g*_1_ *x* + *g*_2_; we can work out (*x*_*c*_, *y*_*c*_) by substituting it in formula (6):

(8) $$\begin{array}{c} \left(1+g_{1}^{2}\right)x^{2}+2g_{1}g_{2}x+g_{2}^{2}-R^{2}=0. \end{array}$$

Solving the coordinates of *C* can be divided into several situations as follows.

*First*. When *x*(*i*) ≠ *x*_0_

coordinates of *C* are as follows:

(9) xc=−2g1g2±(2g1g2)2−4(1+g12)(g22−R2)2(1+g12),yc=g1−2g1g2±(2g1g2)2−4(1+g12)(g22−R2)2(1+g12)+g2,where  g1=yb−y0xb−x0, g2=−yb−y0xb−x0x0+y0;

if *x*_*b*_ < *x*_0_ then

(10) $$\begin{array}{c} x_{c}=\frac{-2g_{1}g_{2}-\sqrt{{\left(2g_{1}g_{2}\right)}^{2}-4\left(1+g_{1}^{2}\right)\left(g_{2}^{2}-R^{2}\right)}}{2\left(1+g_{1}^{2}\right)},\\ y_{c}=g_{1}\frac{-2g_{1}g_{2}-\sqrt{{\left(2g_{1}g_{2}\right)}^{2}-4\left(1+g_{1}^{2}\right)\left(g_{2}^{2}-R^{2}\right)}}{2\left(1+g_{1}^{2}\right)}+g_{2}; \end{array}$$

if *x*_*b*_ > *x*_0_ then

(11) $$\begin{array}{c} x_{c}=\frac{-2g_{1}g_{2}+\sqrt{{\left(2g_{1}g_{2}\right)}^{2}-4\left(1+g_{1}^{2}\right)\left(g_{2}^{2}-R^{2}\right)}}{2\left(1+g_{1}^{2}\right)},\\ {y_{c}=g}_{1}\frac{-2g_{1}g_{2}+\sqrt{{\left(2g_{1}g_{2}\right)}^{2}-4\left(1+g_{1}^{2}\right)\left(g_{2}^{2}-R^{2}\right)}}{2\left(1+g_{1}^{2}\right)}+g_{2}. \end{array}$$

*Second*. When *x*_*b*_ = *x*_0_

 if *y*_*b*_ = *y*_0_ then this is the sink itself; no data needs to be sent;
 if  *y*_*b*_ ≠ *y*_0_  then  *x*_*c*_ = *x*_0_ *x*_*c*_^2^ + *y*_*c*_^2^ = *R* ^2^;
 if  *y*_*b*_ > *y*_0_  then$\;y_{c}=\sqrt{R^{2}-x_{c}^{2}}$;
 if  *y*_*b*_ < *y*_0_  then$\;y_{c}=-\sqrt{R^{2}-x_{c}^{2}}$.

According to coordinate of *C*, the length of line *AC* is

(12) $$\begin{array}{c} \left|A\bar{C}\right|=\sqrt{{\left(x_{c}-x_{0}\right)}^{2}+{\left(y_{c}-y_{0}\right)}^{2}}. \end{array}$$

The length of line *AB* is

(13) $$\begin{array}{c} \left|A\bar{B}\right|=\sqrt{{\left(x_{b}-x_{0}\right)}^{2}+{\left(y_{b}-y_{0}\right)}^{2}}. \end{array}$$

Let *R*_1_ = |*AC*|, *α* = ⌊*R*_1_/*r*⌋, *R*_1_ = *αr* + *b* | *b* ≤ *r*:

(14) D=|AB|=ir+c ∣ i∈{0⋯α},i=⌊Dr⌋, c=D−ir, c∈{0⋯r}.

Data load of *B* is calculated as follows. Its distance from sink is $D=\left|A\bar{B}\right|=ir+c|i\in \left\{0\cdots a\right\},\;x\in \{0\cdots b\}$.

Then check sector area *ℵ* with angle of *dθ* and width of *dx* (see Figure 2). The dimensions of this area are approximately *ℵ*_*s*_ = *D* *dθ* *dx*. The number of nodes in this ring is *ρD* *dθ* *dx*. If it is located in the {*ir* ⋯ *ir* + *b*} | *i* ∈ {0 ⋯ *a*}th ring, that is to say, the location is *D* = *ir* + *c* | *i* ∈ {0 ⋯ *a*}, *c* ∈ {0 ⋯ *b*}, then data load of *ℵ* is as follows.

It is responsible of forwarding all the remote data in sector area whose width is *dx* and is integer multiple of *r* away from *ℵ*. The dimension of these areas can be computed as

(15) dθ((i+1)r+c)dx+dθ((i+2)r+c)dx  +dθ((i+3)r+c)dx+···dθ(ar+c)dx =dθ dx((a−i)c+((i+1+a)(a−i)r2)).

This is the dimension of area *ℵ* which is responsible of forwarding data. Then data load of *ℵ* is

(16) $$\begin{array}{c} d\theta \;dx\left(\left(a-i\right)c+\left(\frac{\left(i+1+a\right)\left(a-i\right)r}{2}\right)\right)\rho . \end{array}$$

Data sent is

(17) {dθ dx((a−i)c+((i+1+a)(a−i)r2)) +dθ(ir+c)dx}ρ.

It can be assumed that the data load is uniformly shared by each node in a very small region. Then data load of each node is

(18) $$\begin{array}{c} \frac{d\theta \;dx\left(\left(a-i\right)c+\left(\left(i+1+a\right)\left(a-i\right)r/2\right)\right)\rho }{d\theta \left(ir+c\right)},\\ dx\;\rho =\frac{\left(\left(a-i\right)c+\;\left(\left(i+1+a\right)\left(a-i\right)r/2\right)\right)}{\left(ir+c\right)}. \end{array}$$

Data sent is {*dθ* *dx*(*ac* + ((1 + *a*)*ar*/2)) + *dθ*(*ir* + *c*)*dx*}*ρ*/*dθ*(*ir* + *c*)*dx*}*ρ* = 1 + ((*a* − *i*)*c* + ((*i* + 1 + *a*)(*a* − *i*)*r*/2))/(*ir* + *c*).

If *D* = *ir* + *c* | *i* ∈ {0 ⋯ *a*},   *c* ∈ {*b* ⋯ *r*} is located in the {*ir* + *b*, *ir* + *r*}th ring data load of *ℵ* can be computed as follows

It is responsible of forwarding all the remote data in sector area whose width is *dx* and is integer multiple of *r* away from *ℵ*. The dimension of these areas can be computed as

(19) dθ((i+1)r+c)dx+dθ((i+2)r+c)dx  +dθ((i+3)r+c)dx+···dθ((a−1)r+c)dx =dθ dx((a−i−1)c+((a−i−1)(a+i)r2)).

Then data received by *ℵ* is

(20) $$\begin{array}{c} d\theta \;dx\left(\left(a-i-1\right)c+\;\left(\frac{\left(a-i-1\right)\left(a+i\right)r}{2}\right)\right)\rho . \end{array}$$

Data sent is

(21) {dθ dx((a−i−1)c+((a−i−1)(a+i)r2)) +dθ c dx}ρ.

It can be assumed that the data load is uniformly shared by each node in a very small region. Then received data of each node is

(22) $$\begin{array}{c} \frac{\left\{\left(a-1-i\right)c+\;\left(\left(a-i-1\right)\left(a+i\right)r/2\right)\right\}}{\left(ir+c\right)}. \end{array}$$

Data sent is

(23) $$\begin{array}{c} \frac{1+\left\{\left(a-1-i\right)c+\;\left(\left(a-i-1\right)\left(a+i\right)r/2\right)\right\}\;}{\left(ir+c\right)}. \end{array}$$

**Figure 3:**
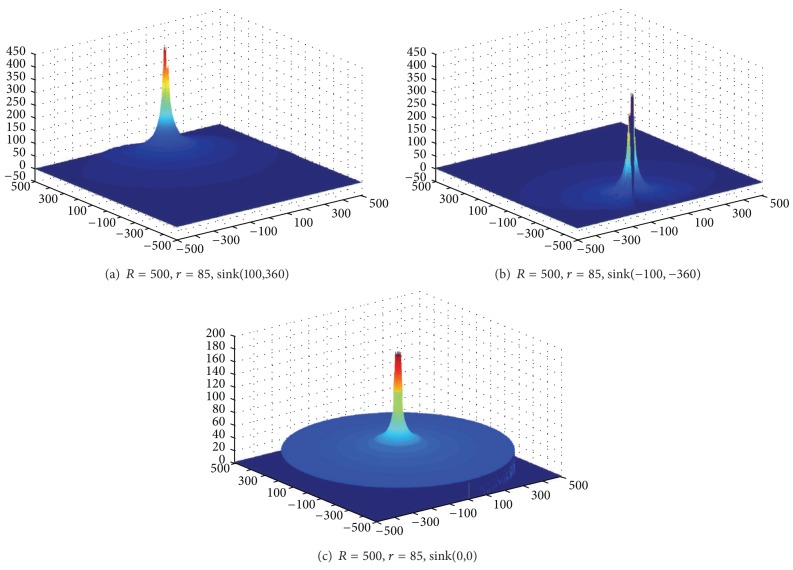
Data load of network.

**Figure 4:**
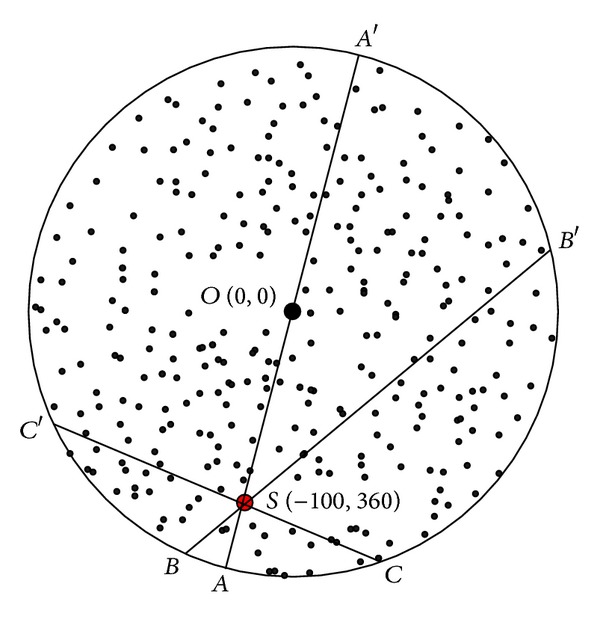
Selected *AA*′, *BB*′, and *CC*′ positions.

Based on Theorem 1, Figure 3 shows the energy consumption map when sink is at different locations. As can be seen from the chart, energy consumption of the network is different when sink is in different locations; nodes near sink do not necessarily suffer higher energy cost. For example, when sink is located near the circumference, power consumption in network-centric side of sink is much higher than the other side near the edge of the network. In order to show more clearly the data load for sink in different orientations and positions, Figure 4 selects three straight lines from the map and Figure 8 shows the statistical chart of the data load on these three lines.

**Figure 5:**
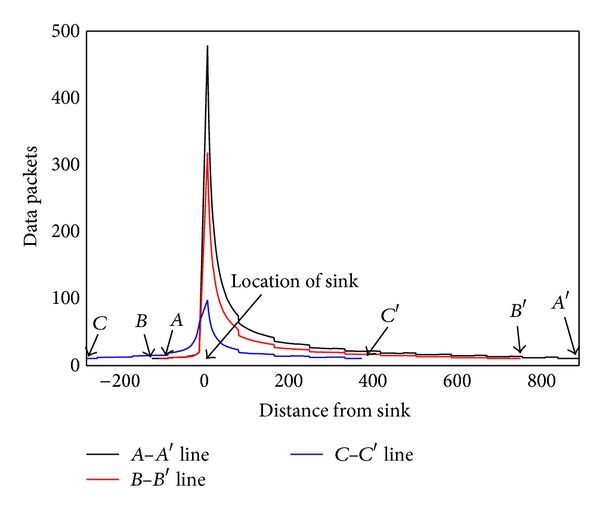
Data load in different distances from sink in different directions.

**Figure 6:**
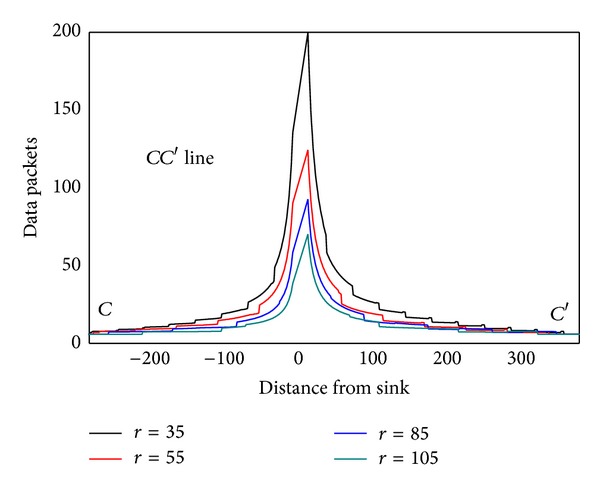
Data load under different communication ranges.

As can be seen from Theorem 1, data load of node is directly proportional to distance (*BC* length) from this node to circumference. The longer *BC* is, the more data node *B* has to bear. It can be seen from Figure 5 that *SA*′ line is the longest, and therefore *SA*′ line has to undertake the most amount of data, but data load of *SA* line on the other side is the smallest, although *SA* is the closest to sink. The situation of *BB*′ and *CC*′ lines is also not the same. This indicates generally the idea that nodes near sink suffer higher volume of data which is wrong. Besides, energy consumption will change with sink location and has close relations to the transmission range.  Figure 6 shows data load of *CC*′ in Figure 4 under different communication range *r*. The general rule is that the bigger *r* is, the smaller the amount of data the node has to bear. The extreme case is when *r* = *R* every node sends data directly to sink. Each node at this time only has to bear one unit of data, which is the least.

**Corollary 2**

Note the transmission range *r* with *f*_*r*_^*i*^(*x*); sink has moved to *A*(*x*_0_, *y*_0_), an arbitrary node *B*(*x*_*b*_, *y*_*b*_); then the energy consumption of node *B* is

(24) $$\begin{array}{c} f_{r}^{i}\left(x\right)=\{\begin{array}{cc} D_{r}E_{\text{elec}}+D_{t}E_{\text{elec}}+D_{t}\varepsilon_{fs}x^{2} & if\;x<d_{0},\;i=0\\ D_{r}E_{\text{elec}}+D_{t}E_{\text{elec}}+D_{t}\varepsilon_{\text{amp}}x^{4} & if\;x\geq d_{0},\;i=0\\ D_{r}E_{\text{elec}}+D_{t}E_{\text{elec}}+D_{t}\varepsilon_{fs}r^{2} & if\;x<d_{0},\;i\neq 0\\ D_{r}E_{\text{elec}}+D_{t}E_{\text{elec}}+D_{t}\varepsilon_{\text{amp}}x^{4} & if\;x\geq d_{0},\;i\neq 0. \end{array} \end{array}$$

**Proof**

According to Theorem 1, the amount of received data of nodes *D* = *ir* + *x* away from the sink is* D*_r_ and the amount of sent data is *D*_*t*_ = *D*_*r*_ + 1. Substituting them into energy formulas (1) and (2) will lead to Corollary 2.

**Figure 7:**
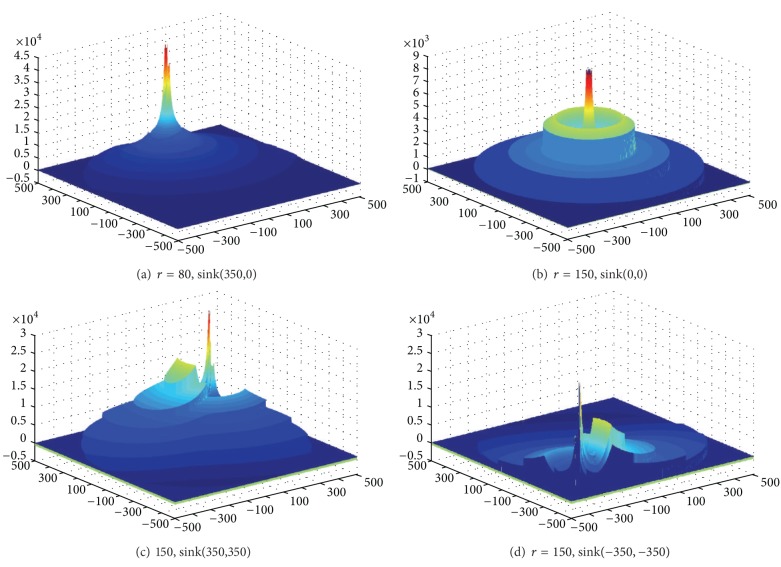
Energy consumption of network (*R* = 500).

Based on Corollary 2, Figure 7 shows the energy consumption under different sink locations and different *r*. As can be seen from the figure, the energy consumption of mobile sink is very complex. So it requires careful planning for moving sink.

**Figure 8:**
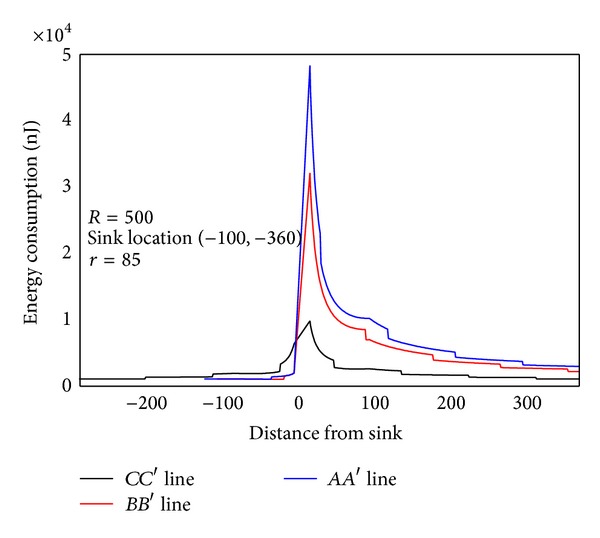
Energy consumption of nodes different distances from the sink in different directions.

**Figure 9:**
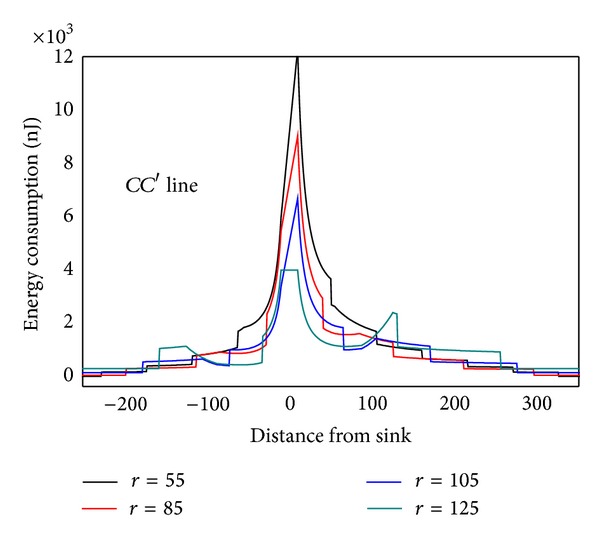
Energy consumption under different communication ranges.

In order to examine the energy consumption of the network in detail, Figure 8 shows energy consumption map of the three lines selected from Figure 4.  Figure 9 shows energy consumption of nodes located at different distance from the sink under various transmission ranges computed through Corollary 2. Different from data load, when node is located at the first *r* distance ring from sink, the transmission range is actually the distance to sink, so it is less than *r*. When distance from node to sink is larger than *r*, transmission range is all the same as *r*. Therefore, the energy consumption does not decline as the distance grows. Its law is that in the first ring where *r* is small, node close to sink has to undertake much higher data load; although the communication range is smaller than *r*, energy consumption rate is still very high. With the increase of *r*, data load declines very fast (see Figure 6), and thus energy consumption of nodes nearest from sink also drops very quickly. Although data load in the edge of first ring is smaller than that within the first ring, the transmission range is larger and hence energy consumption increases. Consequently, there is concave within *r* from sink in the power consumption chart (see Figure 9 energy consumption chart *r* = 125). From the second ring, the communication range is all *r*, but data load is smaller as they are farther from sink, energy consumptions also a decreasing function of distance from sink (see Figure 9). This paper reveals details of network energy consumption for the first time.

### 4.2. The Factor That Affects the Network Lifetime

Data collection strategy for mobile sink is as follows: sink moves to a new location to carry out *k* rounds of data collection and then moves to a new anchor. It goes on like this model until the death of the network. Suppose the total time sink can pause during the survival of the entire network which is Γ. Then goal for sink mobility is to find suitable anchors to achieve the longest life span of the network. And the essence is to maximize the number of rounds of data collection, that is, *Max*⁡(*k* × Γ). As we define the lifetime as an interval till the death of the first node. Therefore, in order to achieve the longest life expectancy, it is necessary to minimize the largest energy consumption among all the nodes.

#### 4.2.1. The Impact of the Number of Anchors

After migrating to the purpose location, sink will conduct data collection. The purpose location is termed as the anchor. The number of locations that sink can pause is termed as anchor number. In fact, the case of static sink can be summed up as the situation where there is only one anchor. From above we know that the center is the best sink location now. In addition, we are able to get the largest network lifetime (equal to static sink). However, when the number of anchor numbers increases to be more than one, the analysis of network life expectancy has become more complex. We assume that sink can move across the whole network, and the anchor number is infinite in theory. But the actual anchor number is limited as follows.

1. Based on the principle of symmetry of a circular network with Proposition  2 (claim 2) in Luo and Hubaux's paper [17] have proven that the track of mobile sink is a ring. That is, trajectory of sink is limited to a circular track within the circle.
2. Because of the limited energy resource of sensor node, the life span of the network is limited as *n* rounds. The sink conducts at least one round of data collection at each anchor; then anchor number is restricted to the *n* points on the circular track. Also, based on the principle of symmetry, these *n* anchor points should be uniformly distributed on the circular trajectory to achieve optimal network performance.
3. The actual physical characteristics of the network, such as sink mobility, network topography, and geography, can limit the anchor sink's stay.

When the sink is located on a track (the track is a ring whose center is at origin; when the radius of ring is *R*_*m*_, it is termed as trajectory or orbit *R*_*m*_) and the anchor location is determined, the algorithm below can compute the energy consumption for each network grid.

**Figure 10:**
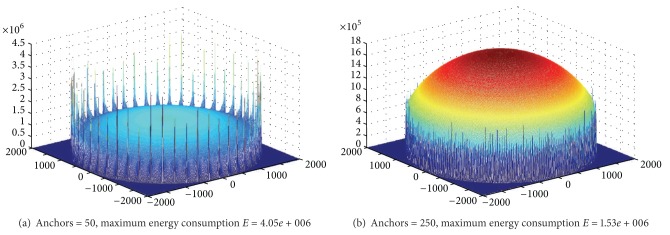
Energy consumption map under different anchor number (*R* = 2000, *R*_*m*_ = 2000 m, and *r* = 85).

Based on Theorem 1, Figure 10(a) shows energy situation of five rounds of data collection for each anchor, and then move to the next location; there is a total of 50 anchors and 250 rounds of data collection. And in Figure 10(b) there is a total of 250 anchors and 250 rounds of data collection. It can be seen that under different anchor numbers, even if the two situations conduct the same rounds of data collection (the same network lifetime), the largest energy consumption in network whose anchor number is bigger is less than half of that in the former network (less anchor number). It indicates that anchor numbers have a greater impact on the network life expectancy. If we define quotient obtained by dividing the maximum energy consumption among all the nodes by the number of rounds of data collection as the network energy efficiency, then the energy efficiency of the network is higher, the higher life expectancy will be under the same initial energy and vice versa.

We also find that network energy efficiency increases with the growth of the anchor. But the efficiency will stay stable after anchor increases to a certain threshold.

The anchor number is mainly affected by the initial energy of node. If the initial energy is small, the life span of the network *n* will be short. However the anchor number cannot exceed *n*, so it will affect the network efficiency when *n* is small. Similarly, the larger the network is, the more energy for one round data collection will be, thus decrease the number of anchor.

Anchor number will not affect the energy efficiency of the network after it rises over certain degree, and the degree is related with the scale of the network. Let the radius of the sink track be *R*_*m*_; study in this paper shows that the degree will be greater when *R*_*m*_ increases and vice versa. The most special situation is that if *R*_*m*_ = 0, then degree of sink is *n* = 0; that is, the anchor number does not affect the network energy efficiency. From the above analysis we can see that anchor number of sink has great impact on network life expectancy. Therefore, previous studies which do not consider anchor number usually simplify the problem.

#### 4.2.2. The Impact of Radius of Mobile Trajectory

**Figure 11:**
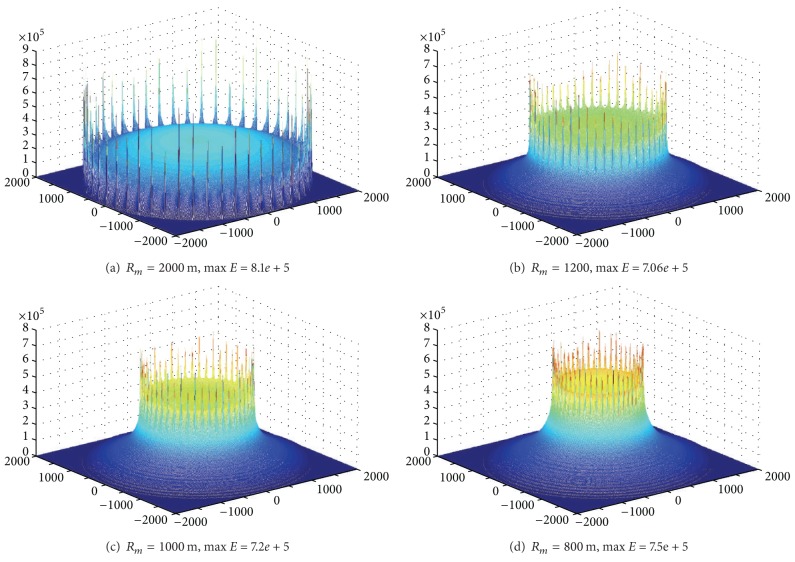
Energy consumption for different values of *R*_*m*_ (*R* = 2000, rounds = 50, anchors = 50, *r* = 85).

Figure 11 shows the energy situation when *R*_*m*_ is, respectively, 2000 m, 1200 m, 1000 m, and 800 m in network with *R* = 2000 m and 50 sink anchors. Largest energy in *R*_*m*_ = 2000 m is not the minimum; instead, *R*_*m*_ = 1200 m has the minimum energy consumption. The above description indicates that the optimal trajectory of mobile sink needs to be carefully optimized and there will be a better route for the longest network life.

#### 4.2.3. The Impact of Communication Range

**Figure 12:**
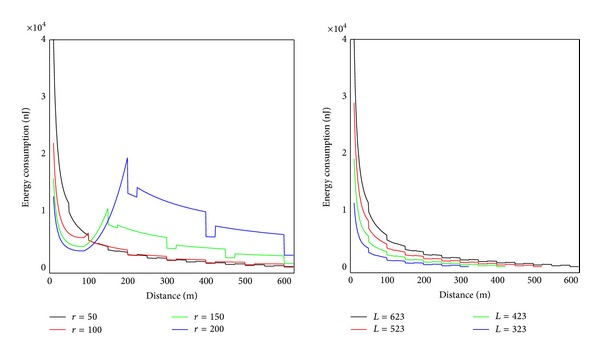
The relationship between energy consumption and *r*.

The communication range *r* has a great impact on the network lifetime.  Figure 12 shows the energy situation of the line which is 620 m from sink to the circumference (similar to |*SA*′ | = 620 m in Figure 4). It can be seen that energy situation changes with different *r*. The rule is as follows: with the increase of *r*, energy consumption of nodes near sink decreases, and energy consumption of nodes far from sink grows. The right chart in Figure 12 shows energy consumption under different length of |*SA*′ | = *L*. The rule is that the greater *L* is, the higher energy consumption will become. Accordingly, the largest lifetime of static sink network is as follows: when *L* = *R*, the value of *r* which can minimize the maximum energy consumption on |*SA*′| is the value that can achieve the largest network lifetime. The energy consumption should be energy cost after one round of data collection. The quotient obtained by dividing node initial energy by the largest energy consumption is the largest network lifetime with static sink.

**Figure 13:**
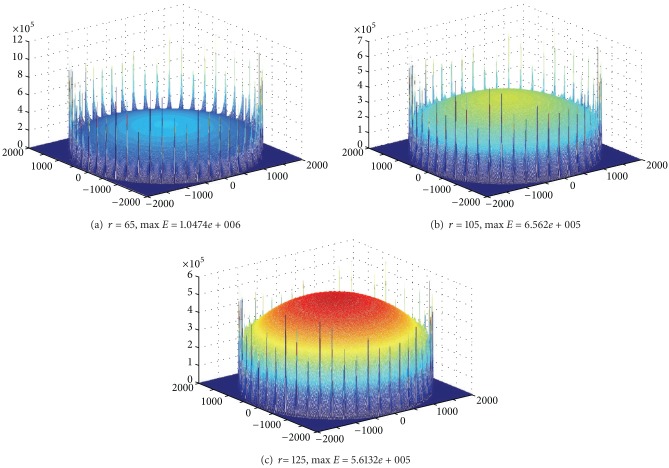
The largest energy consumption of mobile sink network under different *r* (*R* = 2000 m, *R*_*m*_ = 2000 m, rounds = 50, anchors = 50).

Communication range *r* also has a great impact on the lifetime of mobile sink network. We can prove this point through experiment. Below is in the network with *R* = 2000, sink moving along the circumference, and sink pausing anchor should be only 50. Energy consumption in the trajectory is low; energy consumption on the trajectory is high. Therefore, when *r* increases, energy consumption in the trajectory will increase, and energy consumption on the trajectory will reduce. Figures 13(a), 13(b), and 13(c), respectively, show different maximum energy consumption under different *r*. We can see that the difference between network largest energy is nearly doubled when setting different *r* values.

**Figure 14:**
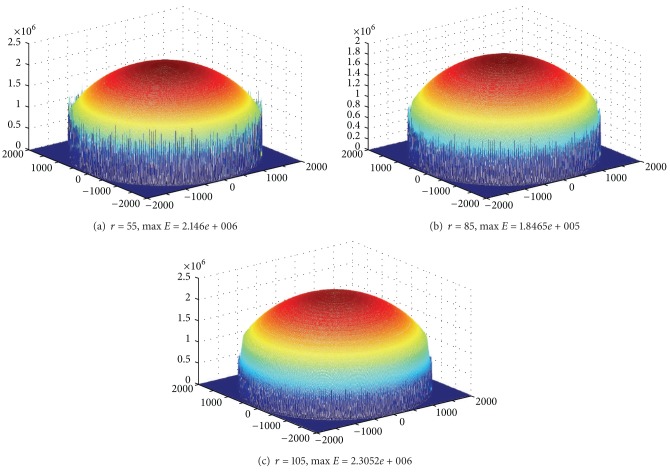
The largest energy consumption of mobile sink network under different *r* (*R* = 2000 m, *R*_*m*_ = 2000 m, rounds = 300, anchors = 300).

Below is in the network with *R* = 2000, sink moving along the circumference, and sink pausing anchor increasing to 300. We can set different *r* values to achieve the smallest largest energy consumption, that is, the maximum network lifetime. Figures 14(a), 14(b), and 14(c) show different maximum energy consumption under different *r*. We can see that network lifetime is also not the same.

**Figure 15:**
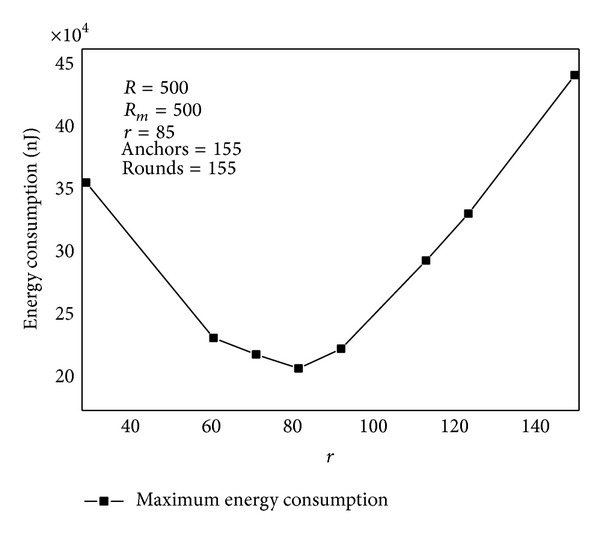
Maximum energy consumption under different *r*.

Figure 15 shows the energy situation after 155 rounds of data collection under different based on Theorem 1. There is a best to obtain the smallest total energy consumption and the largest network lifetime.

**Figure 16:**
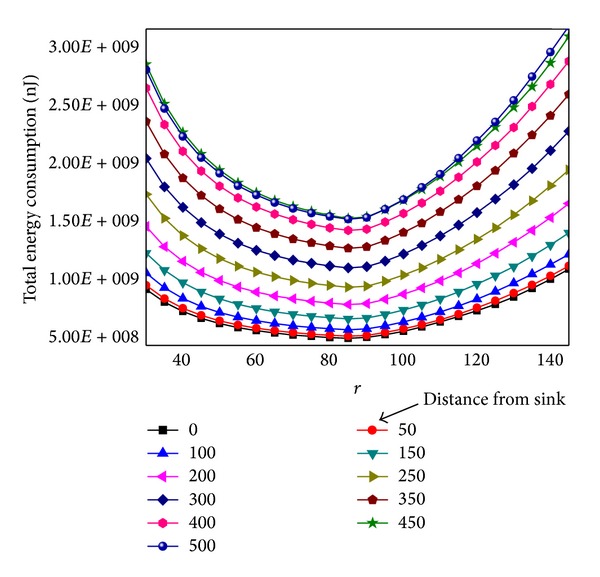
Overall energy consumption under different *r*.

Also *r* is related with the network overall energy consumption.  Figure 16 shows the total energy consumption under different *r* after one round of data collection. The relationship between network total energy consumption and *r* is a concave curve; the concave point minimizes total energy consumption, which is also the best *r* value. The best *r* is not affected by the location of sink. As can be seen from the chart the amount of energy is the least when *r* = 85 m.

## 5. A Distributed Algorithm for Mobile Sink

### 5.1. Distributed Algorithm for Mobile Sink

**Figure 17:**
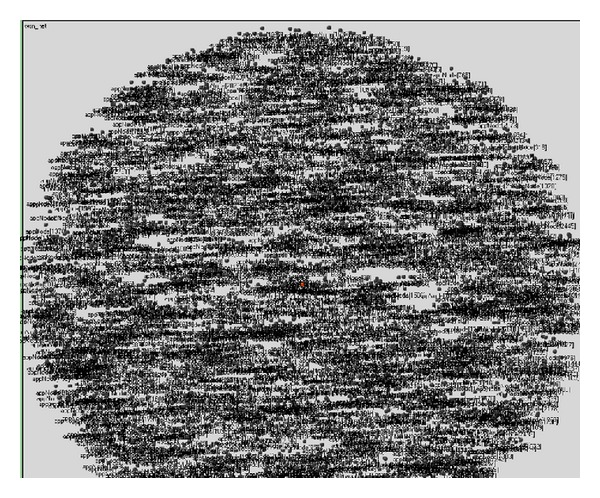
Randomly deployed network.

**Figure 18:**
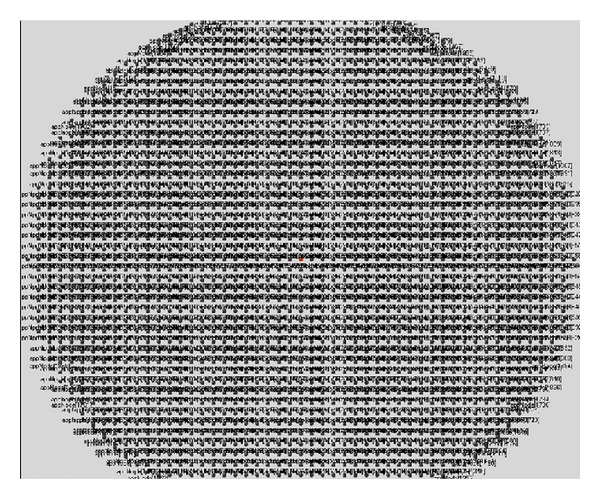
Uniformly deployed network.

**Figure 19:**
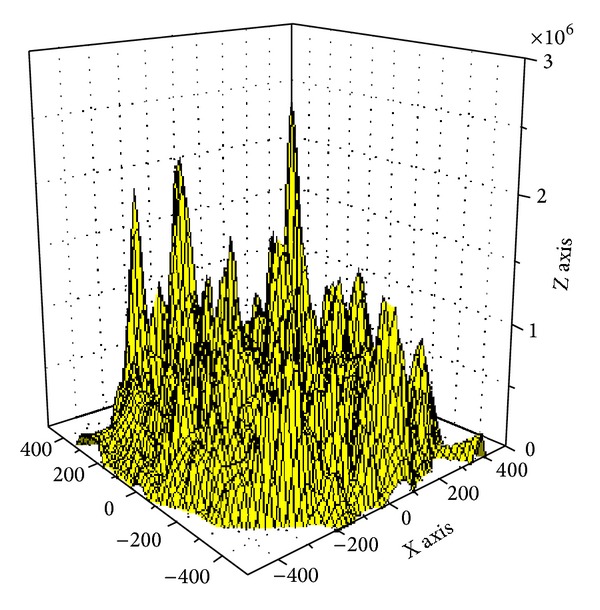
Energy consumption of randomly deployed network.

**Figure 20:**
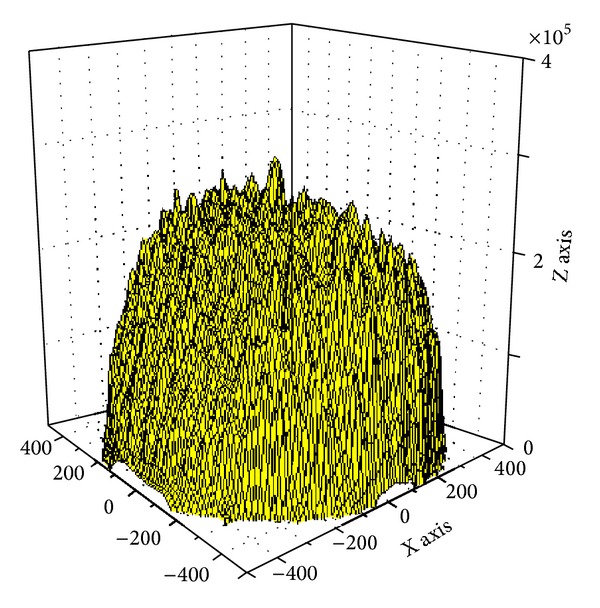
Energy consumption of uniformly deployed network.

In our theoretical calculation nodes are uniformly distributed, and the external environment is homogeneous. But in practice, the deployment of network is always not uniform, and the external environment is not necessarily homogeneous (affected by physical barriers such as restrictions on the terrain). We find in experiments that the deployment of nodes has a great impact on the network lifetime, such as Figures 17 and 18. 3000 nodes are deployed in network with radius of 500 m. In Figure 18 nodes are randomly deployed; in fact, the deployment is uneven. Sparse region will lead to particularly high energy consumption in individual node; these nodes are located at (−104, −387) (76, −20) (−30,279). The energy consumption map is shown in Figure 19.  Figure 20 is an energy consumption diagram of strictly uniform deployment. From observation of experimental results, the trend shown in Figure 19 is in line with the theoretical results. However, it is not as optimal as the results of Figure 20. It can be seen that offline methods cannot adjust sink location according to real world situation. Online distributed algorithm adaptability optimizes the choice of the sink anchors according to the actual energy consumption of the network, so its efficiency is relatively high and adaptable. Therefore, based on the previous theoretical analysis, we propose distributed mobile sink algorithm to avoid the energy hole.

The idea of Algorithm 1 is first to mesh network, then move sink to a grid(*X*_0_, *Y*_0_), calculate the total energy of all the nodes by adding the actual energy consumed and the precalculated theoretical result, and then choose the largest energy* EE* as the* compare energy* for sink at (*X*_0_, *Y*_0_) and repeatedly we switch sink to next grid and calculate its* compare energy*. From all the* compare energies*, we choose the minimum one and set the correspondent sink coordinate as the optimal next pause anchor.

The complexity of Algorithm 1 is *m*∗*n*∗*n*1∗ | *r*|; *m*∗*n* represents the number of grid, *n*1 represents the number of nodes, and |*r*| represents the transmission level of nodes.

### 5.2. Analysis of Mobile Strategy

**Figure 21:**
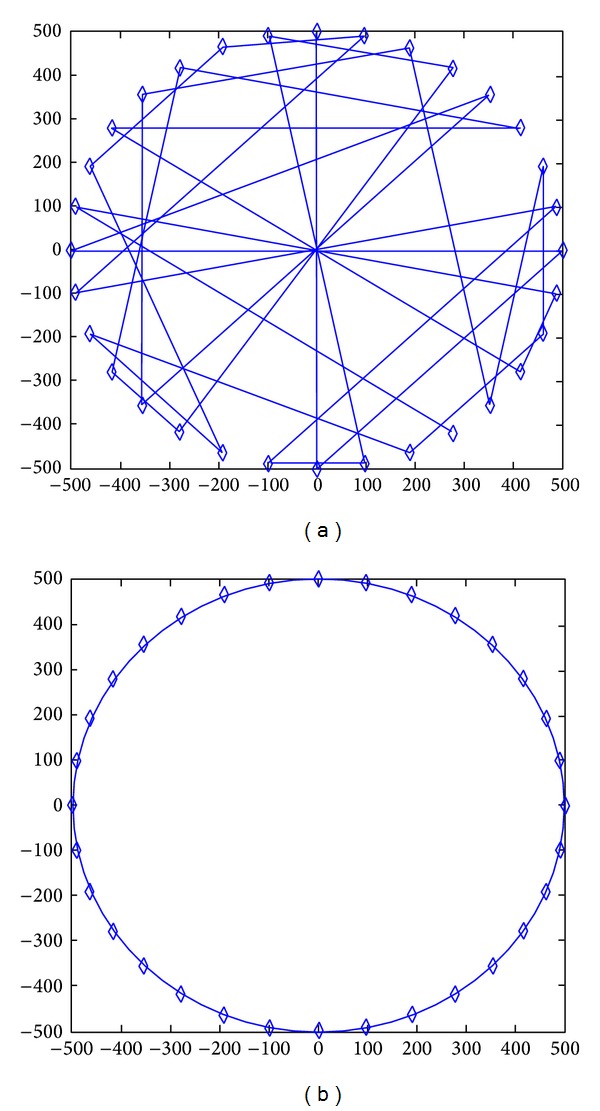
Path for mobile sink.

Algorithm 1 only considers maximizing the life span of the network; sink pausing path is shown in Figure 21(a). However, it fails to discuss the mobility of sink. In fact, it may not be feasible to purely act according to Algorithm 1 in real application. Because the speed of the mobile sink should be slow, sink cannot move around the network without limitations in speed as described in Algorithm 1. In addition, sink mobility will bring extra recourse dissipation and cost; thus the shorter sink moves the better. For these reasons, we recognize that Algorithm 1 can be improved according to the fact that if the total pause of anchors for sink is *N*, then the network life is irrelevant with the sequence of pausing. Therefore, we can precalculate *k*-anchors in Algorithm 1, rather than one single anchor. As long as there is at least one of them located within the scope of current sink migration capability, then ideal moving path of sink is to form a ring around the network. Since the algorithm for calculating *k*-anchors is similar with Algorithm 1, due to space limitations, we omitted it here.

## 6. Performance Analysis and Experimental Results Comparison

This paper applies OMNET++ to carry out experiments; OMNET++ is an open network simulation platform which is open source, component-based and modular for large network and has been widely recognized by the academic community [10]. Experimental parameters are shown in Table  1 from the literature [14], if there is no special note.

### 6.1. Mobile Sink Network Performance Analysis and Experimental Comparison

#### 6.1.1. Impact of the Number of Anchor

**Figure 22:**
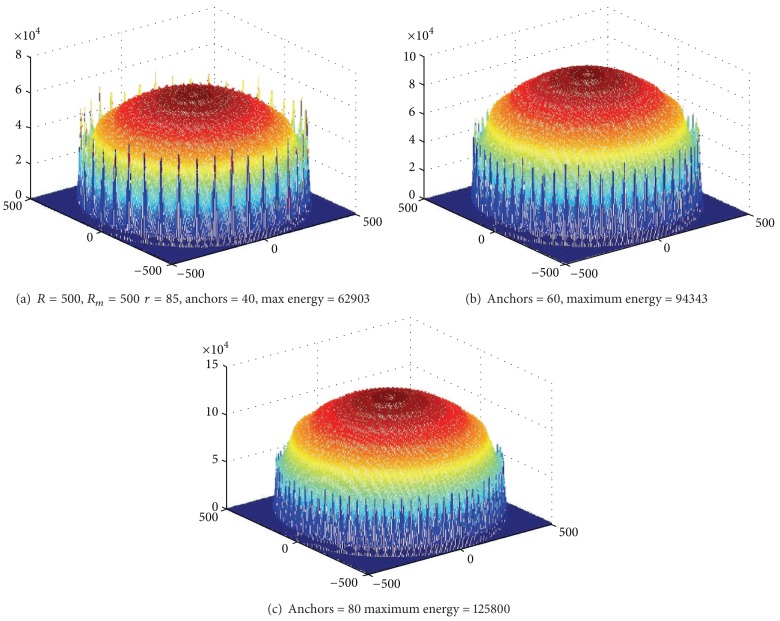
Energy consumption under different numbers of anchor (theoretical value).

**Figure 23:**
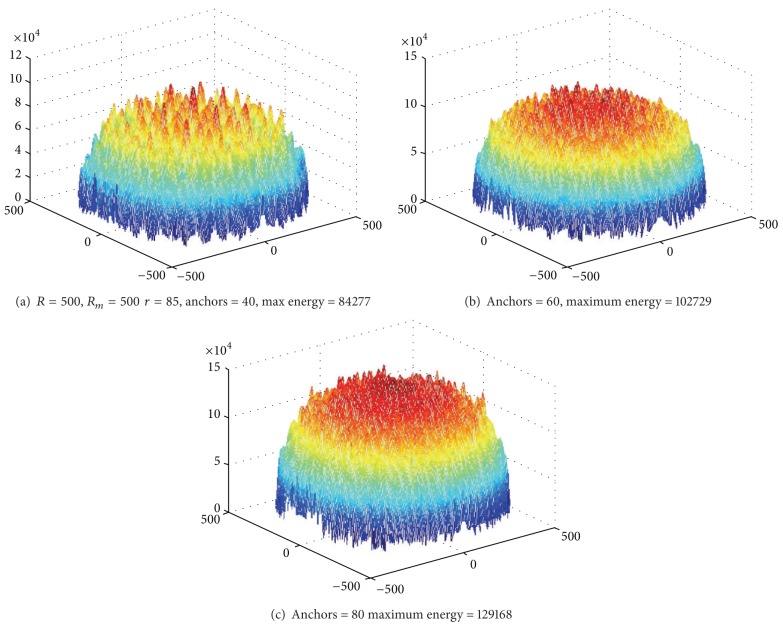
Energy consumption under different numbers of anchor (experimental value).

**Figure 24:**
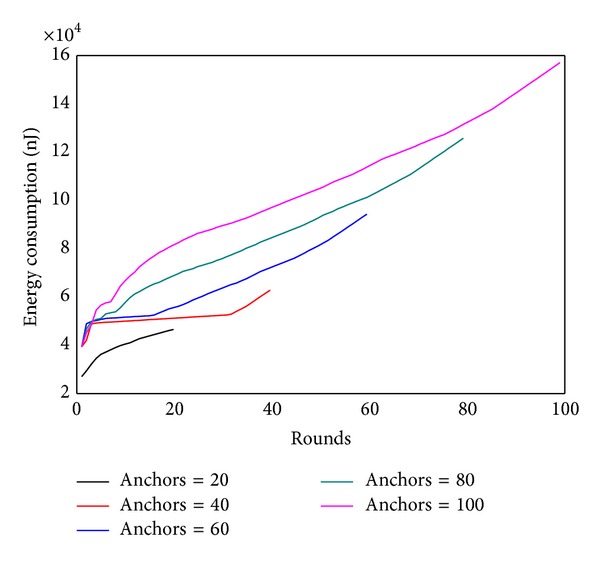
Maximum energy consumption under different numbers of anchor (theoretical value).

**Figure 25:**
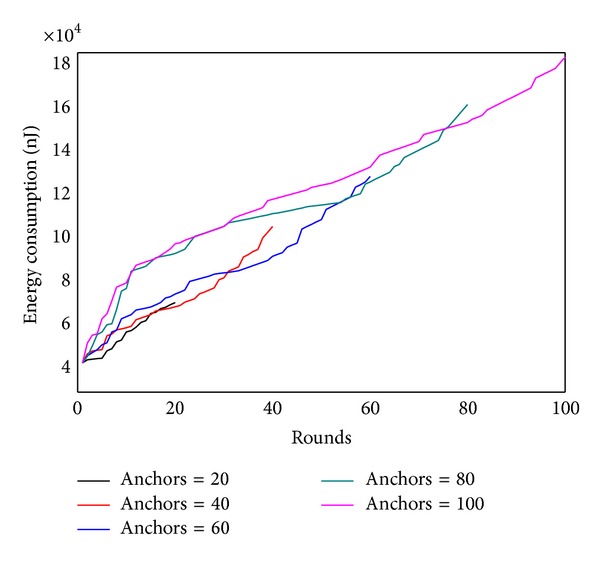
Maximum energy consumption under different numbers of anchor (experimental value).

**Figure 26:**
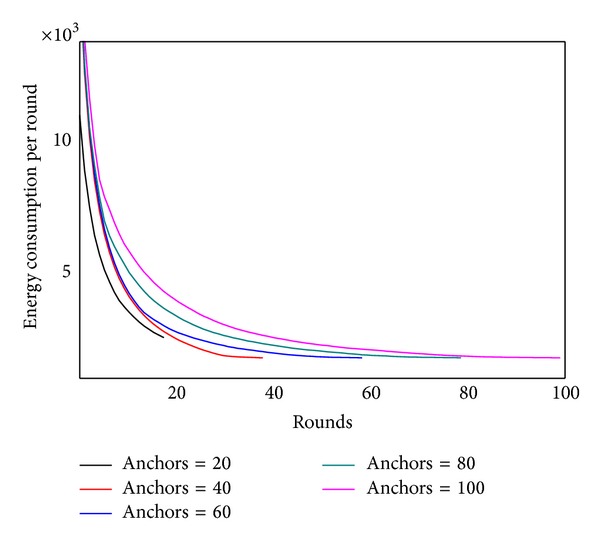
Energy per round under different numbers of anchor (theoretical value).

**Figure 27:**
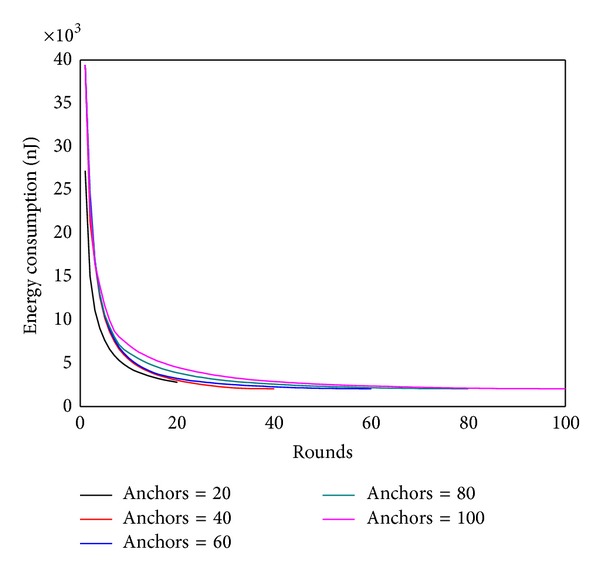
Energy per round under different numbers of anchor (experimental value).

Figures 22 and 23 show, respectively, the theoretical calculations and experimental results of energy consumption under different numbers of anchors. The charts show that theoretical results match the experimental outcome. Figures 24 and 25 compare the maximum energy consumption under different numbers of anchors. The maximum energy consumption is different. We can obtain the* energy per round* by dividing the largest energy consumption by the number of rounds. It is clear that if the* energy per round* is small, then the efficiency of network is high. Figures 26 and 27, respectively, show the* energy per round*. From the chart we can find that the more the anchors are, the better the network efficiency will be. However, after the number of anchor exceeds a certain degree, the network efficiency is stable.

#### 6.1.2. Impact of Transmission Range

**Figure 28:**
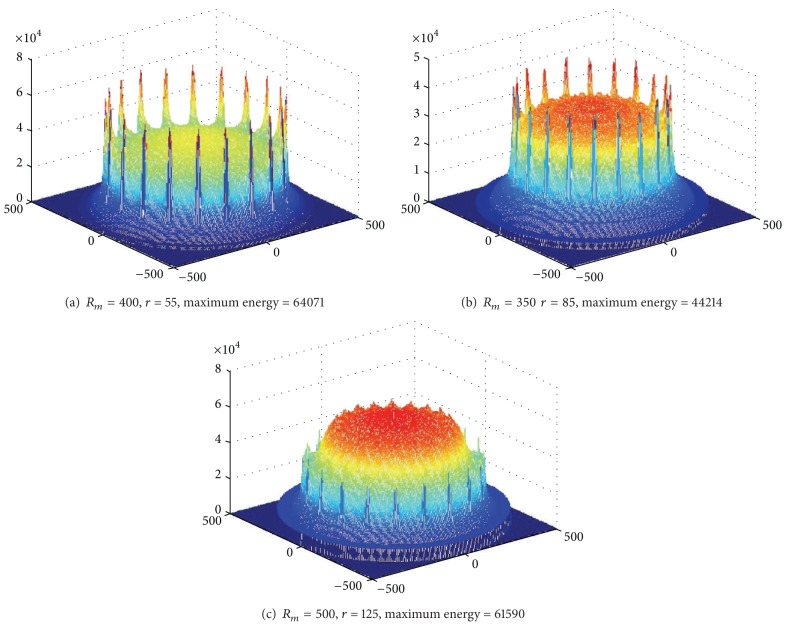
Energy consumption under different transmission range (theoretical value, *R* = 500, rounds = 20, anchors = 20).

**Figure 29:**
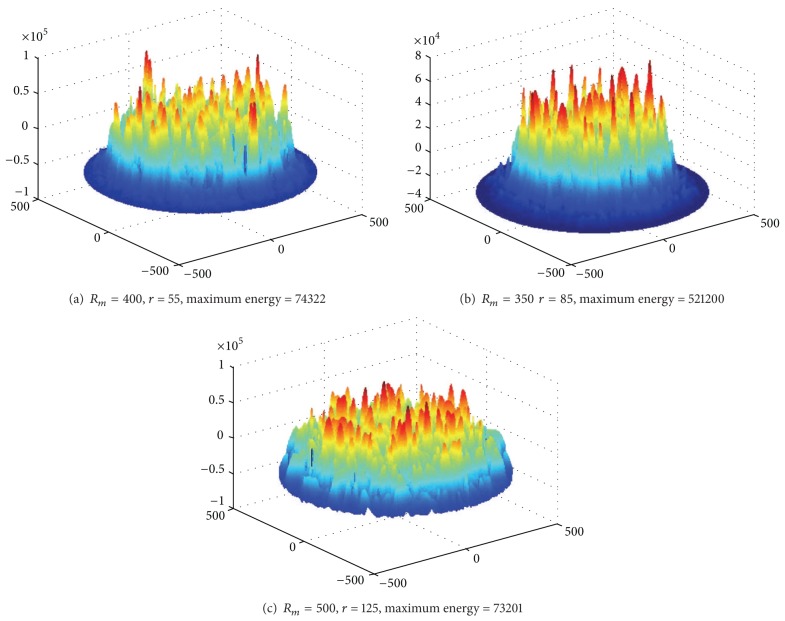
Energy consumption under different transmission range (experimental value, *R* = 500, rounds = 20, anchors = 20).

Figures 28 and 29 show the comparison of network energy dissipation between theoretical analysis and experimental under different transmission ranges and mobile trajectory. We can see a comprehensive impact of *r* and trajectory radius on the network energy consumption and the trend experimental outcomes is very close to the theoretical results.

**Figure 30:**
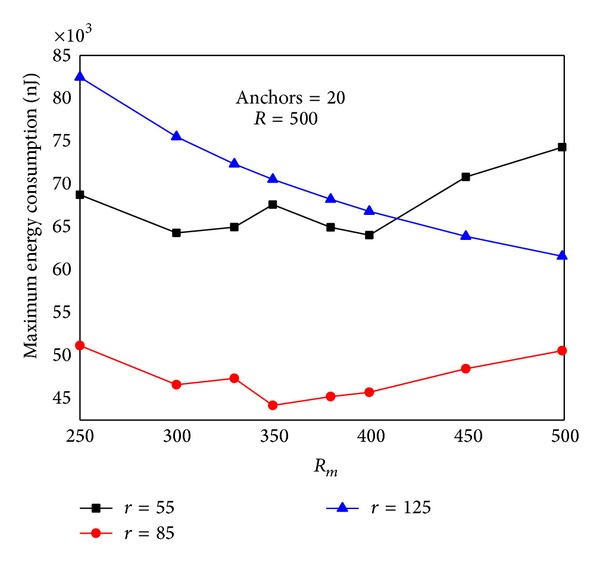
Maximum energy consumption under different transmission range and *R*_*m*_ (theoretical value).

**Figure 31:**
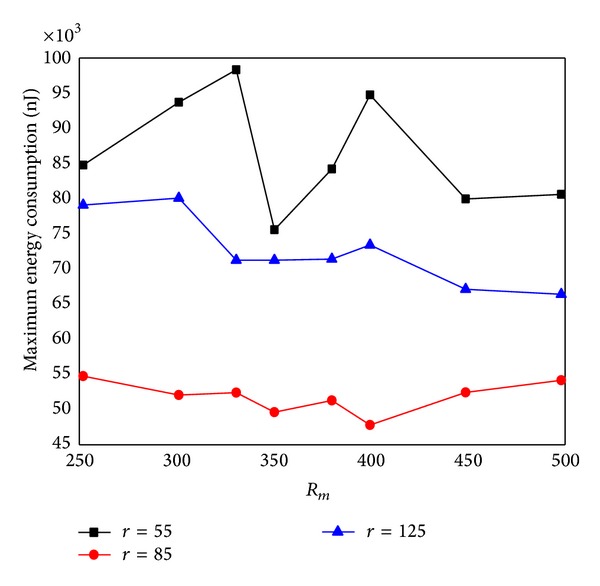
Maximum energy consumption under different transmission range and *R*_*m*_ (experimental value).

Figures 30 and 31 show the maximum energy consumption when sink is on different route. If sink is located in the same trajectory energy consumption can be different for various communication radiuses. If sink is located in distinct trajectory discrepancy between network energy consumption is great. Therefore, optimization of the mobile program requires comprehensive consideration of variety of factors.

**Figure 32:**
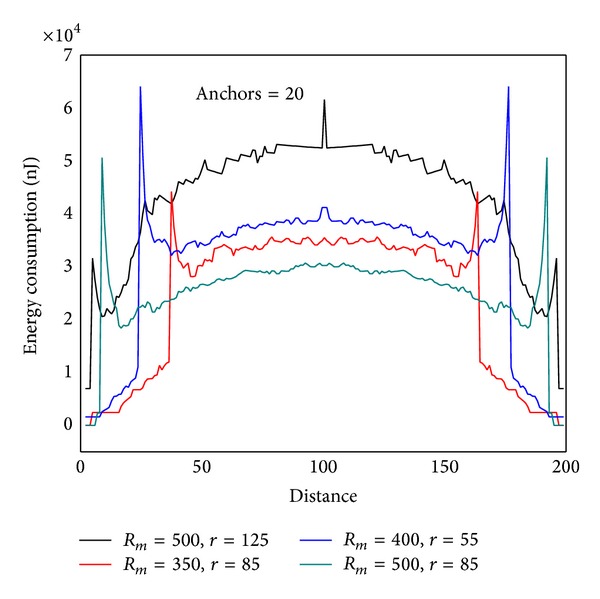
Energy consumption after combining *r* and *R*_*m*_ (theoretical value).

**Figure 33:**
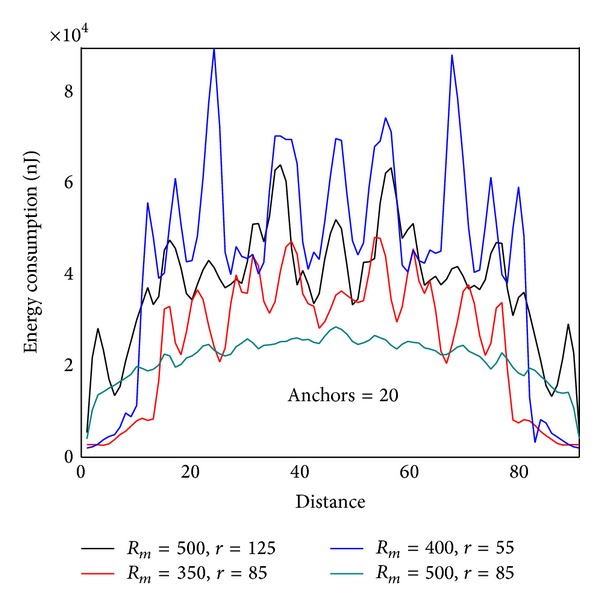
Energy consumption after combining *r* and *R*_*m*_ (experimental value).

Figures 32 and 33 show the energy consumption of nodes on the diameter after 20 rounds of data capture under different* r* and *R*_*m*_. The tendency in experimental result is in line with the theory.

### 6.2. Performance Analysis of the Distributed Algorithm

The experimental scene is as follows: network radius *R* = 500, the numbers of sensor node equal to 3000. Similar with the scene shown in Figure 18, sink collects data for 150 rounds. Based on previous experiments, we know that centralized algorithm in this paper is the same as LUO [17]. We only need to contrast the centralized algorithm, static sink, and distributed algorithm.

**Figure 34:**
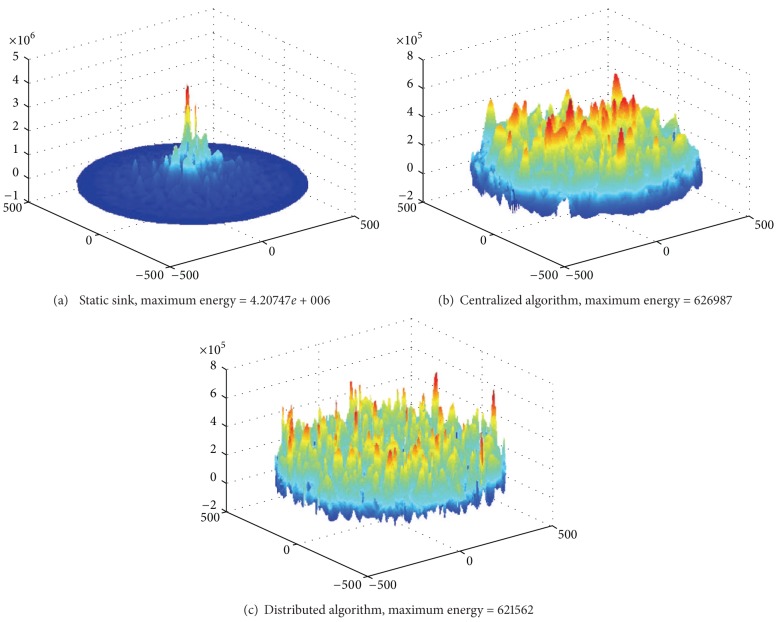
Experimental results comparison of static sink, centralized algorithm, and distributed algorithm.

As theoretical analysis and comparison have been discussed previously, we only compare experimental results here.  Figure 34 shows the energy consumption of these three algorithms after 150 data gathering.

From the experimental results in Figure 34, the energy consumption of static sink is more than the mobile sink approach by nearly an order. The maximum energy cost in distributed algorithm is the smallest. It shows that the proposed algorithm of distributed mobile sink can further improve network lifetime.

**Figure 35:**
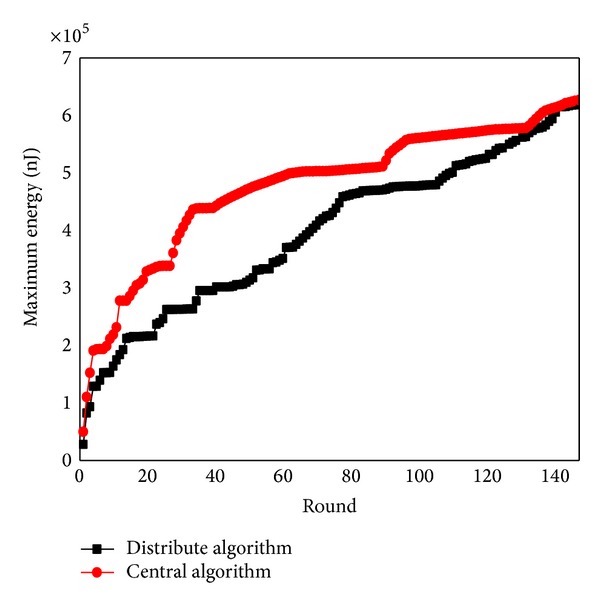
Maximum energy comparisons between centralized algorithm and distributed algorithm.

**Figure 36:**
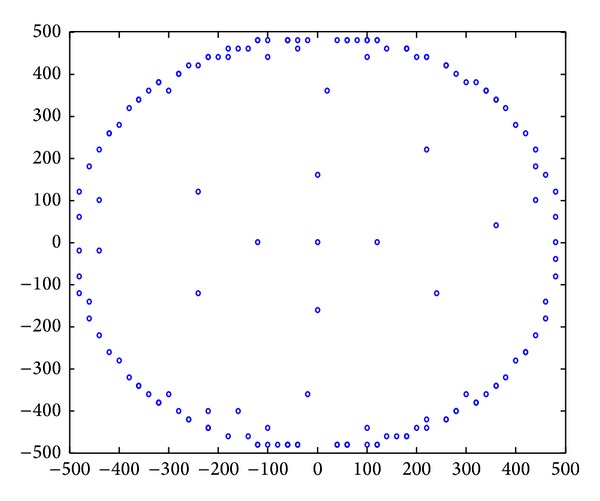
Mobile sink positions in distributed algorithm.

Figure 35 shows the comparison of maximum energy consumption between centralized algorithm and distributed algorithm in the process of data collection.  Figure 36 shows the trajectory of sink computed by distributed algorithm. We can see that the actual optimized track of mobile network is not complete the same as calculated routine. Distributed algorithm has extended the footprint of sink to inside of the network, rather than only moving on the circle.

Distributed algorithm proposed in this paper is superior to all other algorithms as distributed algorithm can adaptively adjust the next location of sink based on actual energy consumption of the entire network. There are similar endeavors in previous studies. This paper can first calculate theoretical energy consumption of the entire network, effectively guiding the sink migration. In contrast with the algorithm which considers energy of the nodes only within one hop of current sink, the algorithm here takes into account the overall energy consumption. Our algorithm is also superior to those algorithms which take area near the sink certainly as hotspot and each time move sink from hotspot to the region with the highest residual energy. Because we can neither simply believe that nodes near sink spend the highest energy consumption, nor that moving sink to the region which remains the highest energy will minimize the maximum energy consumption.

### 6.3. Network Remaining Energy Comparison

**Figure 37:**
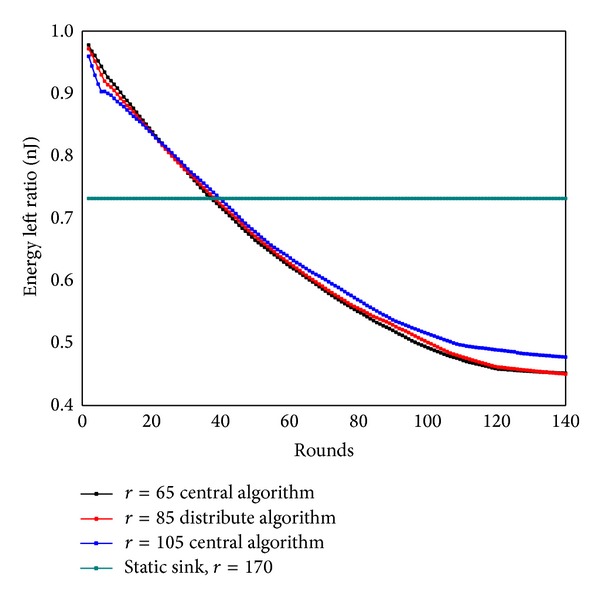
Residual energy ratios under static sink and mobile sink.

**Figure 38:**
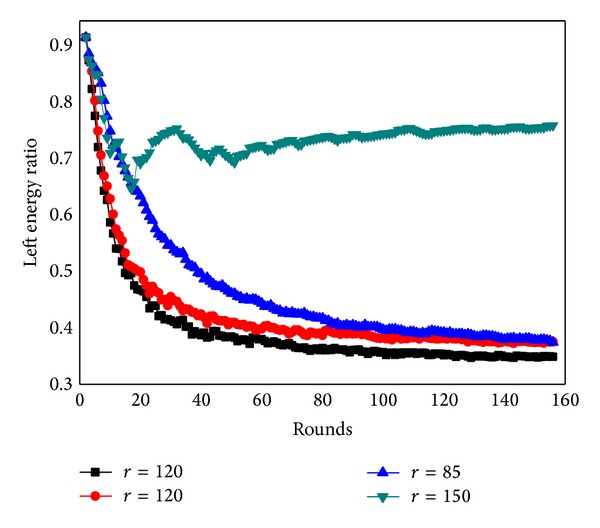
Residual energy ratios under different transmission radius.

Figure 37 shows the residual energy ratio between static sink and mobile sink after 150 rounds of data gathering in network of *R* = 500 m. The remaining energy ratio of static network remains greater than 70 percent all through the run, while the remaining energy ratio of mobile sink strategy drops with the rounds of data collection and finally stays unchanged. Sink mobility has greatly enhanced the energy utilization rate (by 30%).  Figure 38 shows the network remaining energy ratio under different transmission radius and it indicates remaining energy ratios are not the same as communication range changes (residual energy ratio = 1 − (total energy consumption of all the nodes)/(number of nodes × maximum energy consumption)).

We also analyzed the impact of network parameters on the network performance. The node density, from previous study, has little impact on network performance. However, uniformity of the deployment has impact on the network life span. The more uniformly the nodes are deployed, the higher network life expectancy will be. In addition, other factors include the initial nodes energy and the network size. The greater the initial node energy is, the more round it can carry out data collection and vice versa. While increasing the network scale will decrease the network lifetime. As the number of rounds of data collection is equal to the minimum docking anchor of sink node, if the network lifetime is higher, docking anchors will become more and thus energy efficiency increases. Finally, we also experiment on the impact of parameters on the network used in Table 1. Generally, they influence the network performance, however, not the trend.

## 7. Conclusion and Future Works

The main contribution of this paper is as follows: (1) presents a method to accurately calculate energy expenditure of the network when sink is located anywhere; (2) proposes an online distributed algorithm combined with practical sensor networks to achieve better practical significance. In theory analysis and experiment section, we analyze the factors that affect network life in detail. The conclusion is of more general significance.

Although this paper can present a more precise calculation of the energy consumption of the network, the complexity of distributed mobile algorithm is still relatively large. There are several future research works; although there are many theoretical computing for regular network, most of the networks are irregular. Their optimization of mobile sink has more far-reaching applications. Therefore, the next step we plan to extend the energy consumption calculation to general irregular network. Secondly, this paper handles the situation of single mobile sink and multiple mobile sinks, because of its complexity, has so far failed to reach a satisfying method. Based on the conclusion and theoretical calculations in this paper, we will pursue preferable solution for network with multiple mobile sinks [24].

## Figures and Tables

**Algorithm 1:**
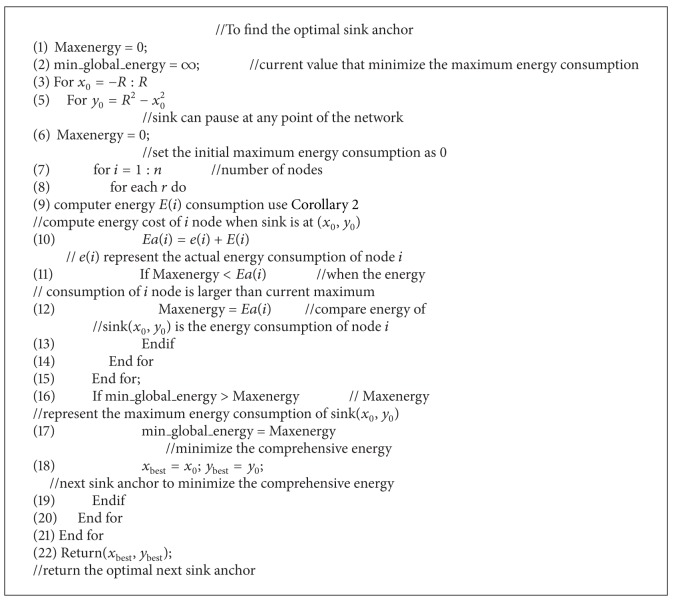
Find best location(*e*(1 : *n*)).
